# Greater Skeletal Muscle Oxidative Capacity Is Associated With Higher Resting Metabolic Rate: Results From the BLSA

**DOI:** 10.1093/geroni/igaa057.409

**Published:** 2020-12-16

**Authors:** Marta Zampino, Richard Semba, Fatemeh Adelnia, Jennifer Schrack, Richard Spencer, Kenneth Fishbein, Eleanor Simonsick, Luigi Ferrucci

**Affiliations:** 1 National Institute on Aging, Bethesda, Maryland, United States; 2 Johns Hopkins University, Baltimore, Maryland, United States; 3 Vanderbilt University Medical Center, Nashville, Tennessee, United States; 4 Johns Hopkins Bloomberg School of Public Health, Baltimore, Maryland, United States

## Abstract

Resting metabolic rate (RMR) tends to decline with aging. The age-trajectory of decline in RMR is similar to changes that occur in muscle mass, muscle strength and fitness. However, while the decline in these phenotypes have been related to changes of mitochondrial function and oxidative capacity, whether lower RMR is associated with poorer mitochondrial oxidative capacity is unknown. In 619 participants of the Baltimore Longitudinal Study of Aging, we analyzed the cross-sectional association between RMR (kcal/day), assessed by indirect calorimetry, and skeletal muscle maximal oxidative phosphorylation capacity, assessed as post-exercise phosphocreatine recovery time constant (tau-PCr), by phosphorous magnetic resonance spectroscopy. Linear regression models were used to evaluate the relationship between tau-PCr and RMR, adjusting for potential confounders. We found that independent of age, sex, lean body mass, muscle density and fat mass, higher RMR was significantly associated with shorter tau-PCr, indicating greater mitochondrial oxidative capacity. In conclusion, higher RMR appears to be associated with a higher mitochondrial oxidative capacity in skeletal muscle. This association may reflect a relationship between better muscle quality and greater mitochondrial health.

